# Schwannoma in the lower inner quadrant of the right breast in a male patient: A rare case report

**DOI:** 10.1097/MD.0000000000041309

**Published:** 2025-01-31

**Authors:** Jingjing Chen, Yan Huang, Haofeng Liu, Yuanyuan Sun, Fufeng Liu

**Affiliations:** a Department of Pathology, Taizhou People’s Hospital of Jiangsu Province, Taizhou, Jiangsu, China; b Department of Pathology, First People’s Hospital of Linping District, Hangzhou, China; c Department of Pharmacy, Suzhou Second People’s Hospital, Suzhou, Anhui, China; d Department of Pharmacy, Suzhou Second People’s Hospital, Suzhou, Anhui, China.

**Keywords:** breast schwannoma, lower inner quadrant, male

## Abstract

**Rationale:**

Lower inner-quadrant breast schwannomas are exceedingly rare, with no reports of their occurrence in male patients in the literature. In this report, we describe a male patient with a schwannoma in the lower inner quadrant of the right breast.

**Patient concerns:**

A 26-year-old man presented at our hospital with a 6-month history of a lump in the right breast and reported pain in the area 3 days prior to presentation. Ultrasonography identified a 0.86 × 0.64 cm hypoechoic nodule 0.41 cm beneath the skin in the lower inner quadrant of the right breast. The nodule exhibited clear boundaries and uniform internal echogenicity with no signs of significant blood flow on color Doppler flow imaging. The patient was concerned that the nodule was benign or malignant.

**Diagnoses:**

Histopathological and immunohistochemical analyses after complete surgical excision confirmed that the lesion was breast schwannoma. The postoperative course was unremarkable and the tumor did not recur during 7 years of follow-up.

**Interventions:**

Biochemical parameters were examined preoperatively. The radiological examination of breast color Doppler ultrasound was performed.

**Outcomes:**

A well-shaped, 0.86 × 0.64 cm mass, complete capsule in the lower inner quadrant of the right breast was surgically resected. The postoperative course was unremarkable and the tumor did not recur during 7 years of follow-up.

**Lessons:**

Breast schwannoma is an extremely rare tumor that is very difficult to preoperatively diagnose. Preoperative biochemical examination and ultrasonography can only provide diagnostic ideas. Histological and immunohistochemical analyses are required for confirmation. It can transform into malignant peripheral nerve sheath tumors, but not often. Consequently, regular postoperative follow-up is required for such patients, especially ultrasonography.

## 1. Introduction

Schwannomas are common benign tumors of the peripheral nerves that originate from Schwann cells, which form the myelin sheath surrounding the nerve cells. While these tumors can occur in any organ, they are most frequently located in the head, neck, and extremities, with schwannomas in the breast being rare. Breast schwannomas account for only 2.6% of all schwannomas. Only 39 cases of the tumor have been reported in the breast by 2019.^[1]^ According to the current case reports, most of the patients are female, and the most common sites are located in the upper outer quadrant. In this study, we present a case of a male patient with schwannoma in the lower inner quadrant of the breast.

## 2. Case report

**Figure 1.**
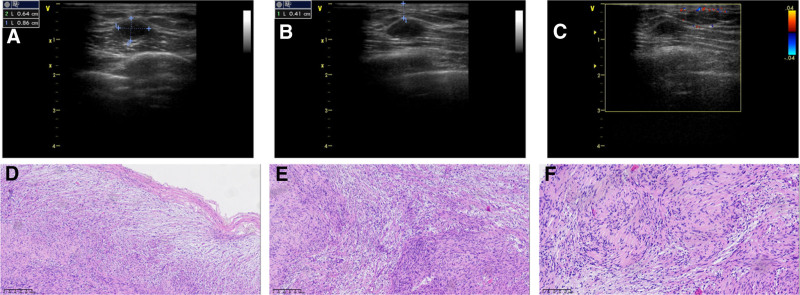
Ultrasound and histopathological examination of the nodules. (A) Ultrasonography shows a hypoechoic nodule with clear boundaries and an oval shape located in the lower quadrant of the right breast. (B) The nodule was located 0.41 cm from the skin. (C) No significant blood flow was observed within the nodules. (D) The tumor had an intact capsule (hematoxylin and eosin staining, ×100). (E) Densely packed spindle cells in the Antoni A area, and a looser Antoni B area (hematoxylin and eosin staining × 100) are shown. (F) Verocay bodies arranged in a palisade pattern are visible in the Antoni A area (hematoxylin and eosin staining × 200).

**Figure 2.**
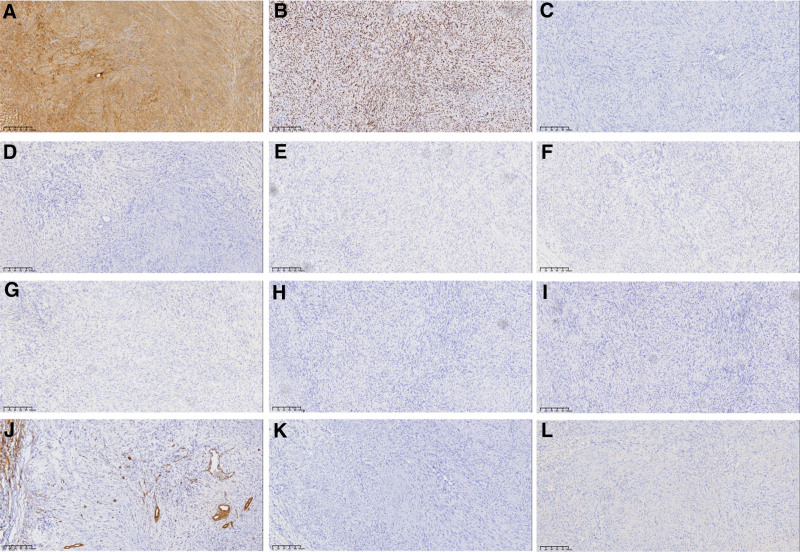
Immunohistochemical analysis. (A) Tumor cells with diffuse and strong positive staining for S-100 (IHC; ×100) are shown. (B) Tumor cells with diffuse and strong positive staining for SOX10 (IHC; ×100) are shown. (C) Less than 2% of the tumor cells were positive for Ki67 (IHC; ×100). (D–L) Tumor cells were negative for the following proteins: CKpan (D), P63 (E), CK5/6 (F), C-erB-2 (G), CD117 (H), DOG1 (I), SMA (J), Desmin (K), and Syn (L) (IHC; ×100). IHC = immunohistochemistry, SMA = smooth muscle actin, Syn = synaptophysin.

A 26-year-old man was admitted to our hospital with a 6-month history of a lump in the right breast that had become painful over the preceding 3 days. According to the patient, the lump size remained relatively unchanged. Physical examination revealed no signs of skin redness, swelling, or nipple discharge. The patient had no signs of von Recklinghausen disease, such as multiple flat café-au-lait spots, skin fold freckling, or visible neurofibromas under the skin.^[2,3]^ Laboratory test results were normal; however, ultrasonography revealed an oval hypoechoic nodule in the lower quadrant of the right breast measuring 0.86 cm × 0.64 cm and located 0.41 cm beneath the skin surface (Fig. 1A, B). The nodule exhibited clear boundaries and uniform internal echogenicity with no signs of significant blood flow on color Doppler flow imaging (Fig. 1C). Breast cancer was mostly painless mass, the appearance of color Doppler ultrasound was irregular, the boundary was not clear, when the metastasis occurred, the axillary lymph nodes were prone to enlargement. Therefore, a benign tumor was considered by color Doppler ultrasound and fibroadenoma was not excluded. The patient underwent complete tumor resection followed by histopathological examination. Low-magnification microscopy revealed a tumor with an intact capsule (Fig. 1D), primarily consisting of Antoni A areas, and the periphery mostly consisting of Antoni B areas (Fig. 1E). Under high magnification, densely packed spindle cells arranged in swirling, weaving, and palisading patterns were observed in Antoni A areas. Additionally, characteristic Verocay bodies comprising 2 parallel rows of tightly arranged nuclei with intervening eosinophilic cytoplasm were scattered throughout the Antoni A area (Fig. 1F). The nuclei in areas A and B were spindle-shaped or oval with no abnormalities, featuring thin membranes, fine and loosely distributed chromatin, and small nucleoli. Immunohistochemical analysis revealed a strong diffuse positivity for S-100 (Fig. 2A), and SOX10 (Fig. 2B) with a Ki67 index <2% (Fig. 2C). The tumor cells were negative for CKpan, P63, CK5/6, C-erB-2, CD117, DOG-1, smooth muscle actin (SMA), Desmin, and synaptophysin (Fig. 2D–L). Based on these histological and immunohistochemical results, a final diagnosis of a right breast schwannoma was made.

## 3. Discussion

Schwannomas arose from Schwann cells in peripheral motor or sympathetic nerves. They most commonly occurred in the head and neck and limbs, whereas schwannomas in the breast were rare.

Breast schwannomas account for only about 0.2 % of all the breast tumors. Studies have shown that the incidences of breast schwannomas are similar in both male and female.^[4]^ The pathogenesis of schwannomas was often associated with NF1.^[2]^ And the latest research suggested that it may also be related to NF2.^[5]^ The pathogenesis of breast schwannomas remained unclear due to their rare incidences.

This study presented the case of a male patient with breast schwannoma, which constitutes only 2.6% of all schwannomas.^[6,7]^ The size of these tumors varies, with estimates ranging from 0.5 to 11 cm (average 3.7 cm, median 3 cm).^[8,9]^ In our case, the maximum diameter was 0.86 cm, representing the smallest male schwannoma reported to the best of our knowledge.^[10,11]^ Most reported cases occur in the upper outer quadrant of the breast; to date, only 1 case of a tumor in the lower inner quadrant has been reported in a female patient.^[12]^ Therefore, this case featuring a schwannoma in the lower inner quadrant of the right breast of a male patient is extremely rare.

Ultrasonographic examination results are valuable for the diagnosis of breast schwannomas. Consistent with previous findings, the ultrasonographic features of the schwannoma in this case included hypoechoic nodules with clear boundaries and no significant blood flow within the nodule, as indicated by color Doppler flow imaging.^[8,13]^ However, a definitive diagnosis requires histopathological examination. Microscopically, the tumor had an intact capsule with alternating dense Antoni A and loose Antoni B areas. In the Antoni A areas, Verocay bodies arranged in a palisade pattern with 2 rows of densely arranged nuclei and intervening eosinophilic cytoplasm were observed.^[14]^ The Antoni B areas showed fewer tumor cells, looser stroma, and myxoid changes than the Antoni A areas.

The breast hamartoma was a mass composed of elements of normal breast tissue, and rare cases showed prominent myoid differentiation, which could lead to spindle cell morphology. The absence of glandular structures within the tumor ruled out hamartoma and gynecomastia.^[15,16]^ Diagnostic features of nodular fasciitis include myxoid stroma, extravasated red cells, and lymphoid cells.^[17]^ The histologic and immunohistochemical findings did not support the diagnosis of nodular fasciitis. The absence of glandular structures within the tumor, along with the immunohistochemical results, excluded metaplastic carcinoma of the breast, leading to a final pathological diagnosis of right breast schwannoma. Gastrointestinal stromal tumors were also excluded because the tumor cells were negative for CD117 and DOG-1, and leiomyoma was ruled out based on the negativity for desmin and SMA. Notably, schwannomas may transform into malignant peripheral nerve sheath tumors (MPNST). Upon loss of additional tumor suppressors (p53) in an NF1 null Schwann cells or Schwann precursor cells, schwannomas can transform into MPNST.^[18]^ Histologically, MPNST diagnosis relies on the identification of necrosis within the tumor tissue, perivascular cell proliferation, spindle cells with hyperchromatic nuclei, and a marked increase in mitotic figures.^[19]^ Histological examination in this case did not reveal evidence for MPNST. Complete surgical resection is the preferred treatment for schwannomas; however, in cases of MPNST, a sufficiently wide margin (at least 2 cm) is recommended in addition to complete surgical resection. Although the prognosis of schwannoma was good, annual follow-up was necessary.^[20]^ In this case, the patient recovered well postoperatively with unremarkable follow-up of 7 years.

In conclusion, this case demonstrates that, although they are extremely rare, schwannomas in the lower inner quadrant of the breast should be considered in the differential diagnosis of breast masses in men.

## Acknowledgments

The assistance provided by the clinicians in this case is greatly appreciated.

## Author contributions

**Conceptualization:** Yan Huang, Haofeng Liu, Yuanyuan Sun.

**Data curation:** Yan Huang.

**Formal analysis:** Jingjing Chen, Yan Huang, Yuanyuan Sun.

**Funding acquisition:** Fufeng Liu.

**Methodology:** Jingjing Chen, Haofeng Liu.

**Software:** Haofeng Liu.

**Writing – original draft:** Jingjing Chen.

**Writing – review & editing:** Fufeng Liu.
